# A Fusion Protein of RGD4C and β-Lactamase Has a Favorable Targeting Effect in Its Use in Antibody Directed Enzyme Prodrug Therapy

**DOI:** 10.3390/ijms16059625

**Published:** 2015-04-28

**Authors:** Hao Wang, Xiao-Liang Zhou, Wei Long, Jin-Jian Liu, Fei-Yue Fan

**Affiliations:** Tianjin Key Lab of Molecular Nuclear Medicine, Institute of Radiation Medicine, Chinese Academy of Medical Sciences and Peking Union Medical College, Tianjin 300192, China; E-Mails: wanghao@irm-cams.ac.cn (H.W.); zhouxiaoliang@irm-cams.ac.cn (X.-L.Z.); longway@irm-cams.ac.cn (W.L.); liujinjian@irm-cams.ac.cn (J.-J.L.)

**Keywords:** antibody directed enzyme prodrug therapy, RGD4C, β-lactamase, targeting

## Abstract

Antibody directed enzyme prodrug therapy (ADEPT) utilizing β-lactamase is a promising treatment strategy to enhance the therapeutic effect and safety of cytotoxic agents. In this method, a conjugate (antibody-β-lactamase fusion protein) is employed to precisely activate nontoxic cephalosporin prodrugs at the tumor site. A major obstacle to the clinical translation of this method, however, is the low catalytic activity and high immunogenicity of the wild-type enzymes. To overcome this challenge, we fused a cyclic decapeptide (RGD4C) targeting to the integrin with a β-lactamase variant with reduced immunogenicity which retains acceptable catalytic activity for prodrug hydrolysis. Here, we made a further investigation on its targeting effect and pharmacokinetic properties, the results demonstrated that the fusion protein retains a targeting effect on integrin positive cells and has acceptable pharmacokinetic characteristics, which benefits its use in ADEPT.

## 1. Introduction

Antibody directed enzyme prodrug therapy (ADEPT) is a targeted therapeutic method based on antibody-enzyme conjugates and prodrugs [1,2], which is a promising approach for targeting treatment of cancer. It is designed to restrict the action of cytotoxic agents at the tumor sites. The principle of ADEPT is to use an antibody targeted to a tumor-associated antigen to convey an enzyme to the tumor site. A nontoxic prodrug, a substrate for the enzyme, is administrated after eliminating of the unbound antibody-enzyme conjugate from blood and normal tissues, and cleaved by the enzyme, then a potent cytotoxic agent is generated [1,3]. The cytotoxic agent can be precisely released at tumor site which can avoid systemic toxicity, and can diffuse into adjacent cells making the non-antigen expressed tumor cells undergo treatment [4], which can further improve the treatment. The enzymes used in this strategy have relatively fixed relations with prodrugs, such as carboxypeptidase G2 and benzoic mustard [5], nitroreductase and CB1954 [6,7], l-methioninase and selenomethionine [8,9], cytosine deaminase and 5-fluorocytosine [9,10], β-lactamase and cephalosporin prodrugs [11,12]. In the previous study we constructed a conjugate (RGD4CβL) of RGD4C (ACDCRGDCFCG) and β-lactamase variant with low immunogenicity for use in the enzyme prodrug therapy, in which the RGD4C motif served as direct group for its specificity to α_v_β_3_ integrin which is overexpressed on tumor cells and is usability for incorporation into proteins by recombinant technology [13,14,15], and studies showed the fusion protein not only retains catalytic activity of β-lactamase but has low immunogenicity and high stability [16,17]. The targeting and pharmacokinetics of the conjugate plays a key role in ADEPT. Therefore, we investigated the targeting effects and pharmacokinetics of the RGD4CβL in the present study, the results revealed a normal cell binding manner *in vitro* and favorable distribution and elimination mode *in vivo*, which benefits its use in enzyme prodrug therapy.

## 2. Results and Discussion

### 2.1. Immunofluorescent Staining

**Figure 1:**
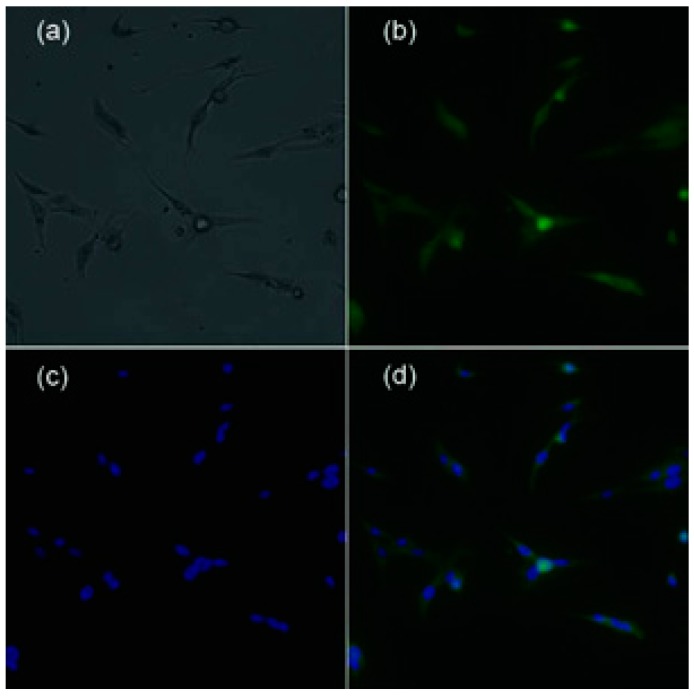
Fluorescent images of FITC-RGD4CβL stained C6. (**a**) C6, white light; (**b**) FITC-RGD4CβL, fluorescence of FITC; (**c**) DAPI; (**d**) overlay of (**b**) and (**c**). Immunofluorescent staining showed that the RGD4CβL adhered to the C6 cells, which indicated the normal binding of RGD4C motif.

To confirm its affinity on tumor cells, the RGD4CβL was labeled with fluorescein isothiocyanate (FITC) obtaining the labeled product (FITC-RGD4CβL) and immunofluorescent staining was performed. The concentration of FITC-RGD4CβL, which was determined by [(A280 − 0.31 × A495)/1.4], was 0.73 mg/mL; the ratio of FITC to RGD4CβL, which was determined by [3.1 × A495/(A280 − 0.31 × A495)], was 6.3; and the FITC-RGD4CβL was diluted twice and used for staining, and the nucleus was stained using 4',6-diamidino-2-phenylindole (DAPI). The fluorescent images are shown in Figure 1.

### 2.2. Radiolabeling and Radiochemical Purity

**Figure 2:**
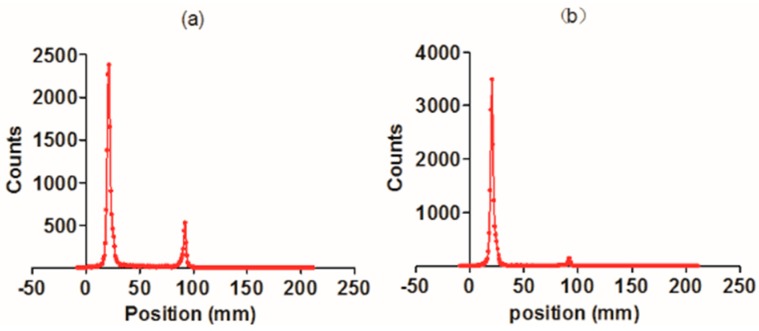
Radiolabeling efficiency and the purity of the ^99m^Tc-RGD4CβL. The crude product was separated on silica gel plate. The main product had a retention factor (RF) of 0.17 and the RF of [^99m^Tc(H_2_O)_3_(CO)_3_]^+^ was 0.46. Labeling efficiency was calculated to be 82.6% (**a**); After purification by ultrafiltration, the radiochemical purity of the ^99m^Tc-RGD4CβL was 98.7% (**b**).

The RGD4CβL was labeled with ^99m^Tc and purified, and then the labeling efficiency and radiochemical purity were determined using thin layer chromatography. The ^99m^Tc-RGD4CβL and the unbound [^99m^Tc(H_2_O)_3_(CO)_3_]^+^ were separated well on silica gel plates, using acetone as the mobile phase. In the present experiment, the radiolabeling efficiency as determined using thin layer chromatography, was 82.6% (Figure 2a). The crude product was purified by ultrafiltration to obtain the final product with radiochemical purity of 98.7% (Figure 2b).

### 2.3. In Vitro Stability

**Figure 3:**
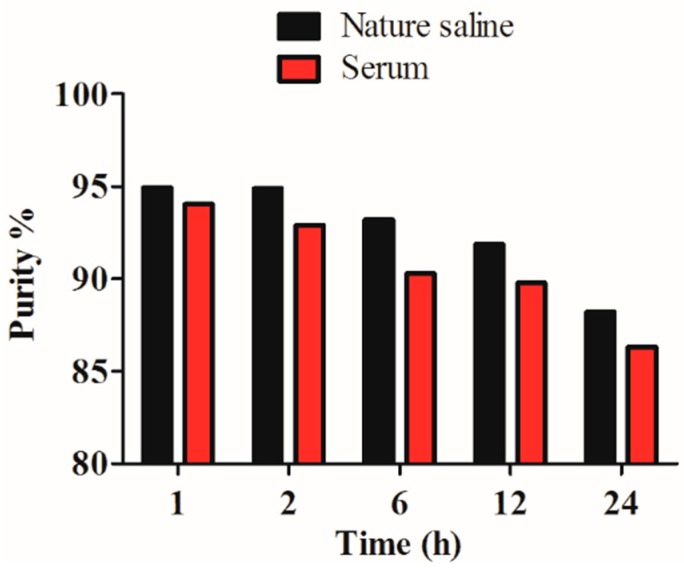
*In vitro* stability. Radiochemical purity of the ^99m^Tc-RGD4CβL remained more than 88% and 86% periodically over 24 h in normal saline at room temperature (25 °C) and in human serum at 37 °C, which was favorable for *in vivo* use.

The radiochemical purity of the ^99m^Tc-RGD4CβL was assayed under two conditions. Under both conditions the ^99m^Tc-RGD4CβL showed good stability (Figure 3).

### 2.4. In Vitro Evaluation of the ^99m^Tc-RGD4CβL

**Figure 4:**
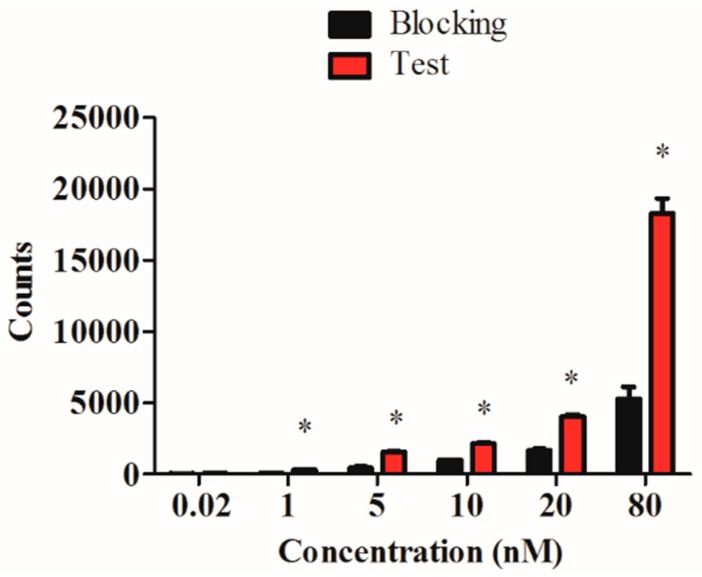
Specific binding of the ^99m^Tc-RGD4CβL. The ^99m^Tc-RGD4CβL was incubated with C6 cells at a concentration of 0.02 to 80 nM, which showed normal binding manner, when the cells were treated with cold protein, the binding was blocked. Data were analyzed using Student’s *t*-test, and asterisks represent statistically significant values (*p* < 0.05). The result showed a specific binding manner of ^99m^Tc-RGD4CβL, indicated normal affinity of RGD4C as a direct motif.

The carbonyl technetium was conjugated to the His-tag, which is far from the activity center; so the affinity and specificity of the RGD4CβL may remain unaffected. To confirm this hypothesis, an *in vitro* binding assay was performed using C6 cells, and the result showed a normal binding manner that could be blocked with cold protein (Figure 4).

### 2.5. Blood Clearance of the ^99m^Tc-RGD4CβL

**Figure 5:**
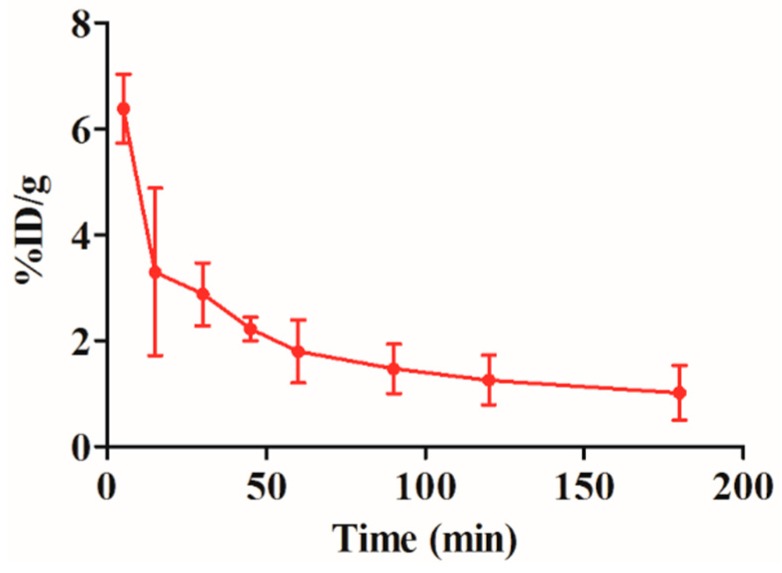
Blood clearance of the ^99m^Tc-RGD4CβL. Three Wistar rats were injected with the ^99m^Tc-RGD4CβL. Blood was drawn at different time-points, and radioactivities were measured by a gamma counter, data are shown as %ID/g, T_1/2_α and T_1/2_β were 7.8 and 21.9 min respectively.

The pharmacokinetics of conjugate is important for its *in vivo* use; thus, we investigated the metabolism of the ^99m^Tc-RGD4CβL in rats. The radioactivity-time curve shown below, the blood clearance was fast during the first 50 min. The half-lives of distribution (T_1/2_α) and elimination (T_1/2_β) were 7.8 and 21.9 min respectively (Figure 5). The short half-lives were acceptable for its use in ADEPT.

### 2.6. Biodistribution

**Figure 6:**
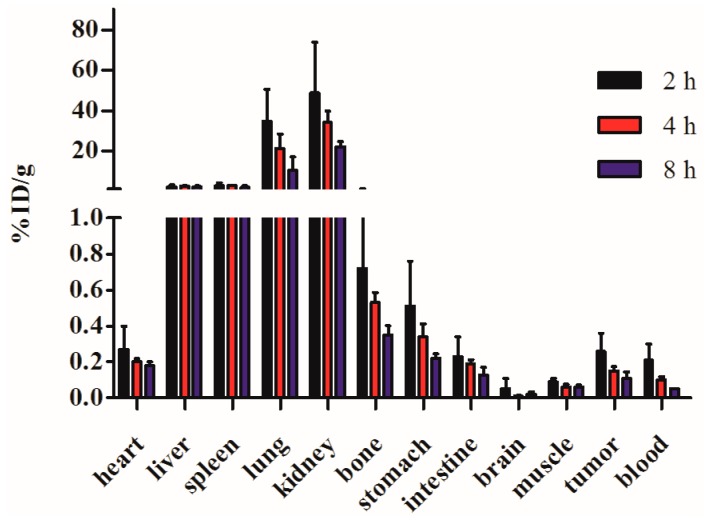
Biodistribution of the ^99m^Tc-RGD4CβL in xenograft-bearing rats. Data are shown as %ID/g, and expressed as mean ± SD (*n* = 4). High uptake and slow decline in tumor relative to blood was observed. The radiolabeled protein was mainly metabolized through the kidney, which may cause the high uptake. High uptake in liver, spleen and lung may be caused due to the dissociation of the radio-nuclide from the ^99m^Tc-RGD4CβL. The high uptake in tumor than background may be favorable for its use in ADEPT.

Rats bearing C6 xenografts were injected with the ^99m^Tc-RGD4CβL, and dissected at 2, 4, and 8 h. The radioactivities of different organs were measured with a gamma counter. It was noted that the radiolabeled protein was mainly metabolized through the kidney. The radioactivity in tumor showed a slower decline than in blood, which benefits its use in enzyme prodrug therapy (Figure 6).

ADEPT combining the high affinity and specificity of monoclonal antibodies and high catalytic activity of enzymes, which can restrict the action of cytotoxic drugs into tumor site, has become a promising approach for tumor treatment and derived a variety of related modalities [18]. Conjugates used in enzyme prodrug therapy, which consist by enzymes coupled with targeting molecules, require certain characteristics such as good stability, low immunogenicity, ease of manufacturing, and no substrates or inhibitors in human body. The RGD4CβL studied in the present work which was composed of RGD4C and broadly targeted multiple tumors overexpressing integrin and β-lactamase variant. This is an efficient hydrolase for cephalosporin prodrugs that has been manufactured in previous work and has shown high catalytic efficacy and low immunogenicity which benefits its use in enzyme prodrug therapy. Its targeting effect and pharmacokinetic properties were investigated with ^99m^Tc labeling in this work to confirm its applicability. Here, we observed that the RGD4CβL could be efficiently labeled with ^99m^Tc, with full retention of it functionality (*i.e.*, specific recognition of α_v_β_3_ integrin). Immunofluorescent staining of FITC-RGD4CβL and binding assay of ^99m^Tc-RGD4CβL confirmed its affinity and specificity on tumor cells. Good *in vivo* tumor-to-background ratios were already obtained at 2 h after injection of the ^99m^Tc-RGD4CβL, which revealed the rapid elimination of unbound conjugate from the circulation. The ^99m^Tc-RGD4CβL is rapidly cleared from the blood, mainly via the kidneys. This property agreed the typical behaviour of peptides and small proteins whose molecular weight is below the threshold that can be filtered by the glomerular membrane [19]. Meanwhile, ^99m^Tc-RGD4CβL was eliminated relatively slow in tumor compared with rapid clearance from blood, which means that the prodrug can be administrated when the fusion protein eliminated from normal tissues and the residual in tumor site can release the cephalosporin prodrug to kill tumor cells.

In summary, the conjugate (RGD4CβL) can be labeled with ^99m^Tc efficiently, retaining the affinity and specificity. The pharmacokinetic property of the radiolabeled conjugate was codetermined by the size, the radionuclide and the affinity to tumor cells, which was favorable for its *in vivo* use. The High uptake and slow decline in tumor opened a perspective towards antibody-targeted imaging combined chemotherapy for optimization of dose and time schedules. Future studies will be performed in accordance with this combination concept to reveal the potential usability of RGD4CβL in ADEPT and molecular imaging.

## 3. Experimental Section

### 3.1. Materials

Fresh ^99m^Tc-pertechnetate eluent was obtained from a ^99^Mo–^99m^Tc radionuclide generator (China Institute of Atomic Energy). Sodium borohydride, sodium carbonate, HCl, sodium potassium tartrate tetrahydrate and fluorescent dyes (FITC and DAPI) were purchased from Sigma-Aldrich (St. Louis, MO, USA). Silica gel plates were purchased from Yantai Jiangyou silica Ltd. (Shandong, China). Carbon monoxide and carbon dioxide were purchased from Tianjin kunteng gas Ltd. (Tianjin, China). Centrifugal filter units with a molecular weight cut-off of 3000 Da were purchased from Millipore (Bedford, MA, USA), and used according to the specifications. Medium, fetal bovine serum (FBS), penicillin, streptomycin was purchased from Hyclone (Logan, UT, USA). Human serum (a mixture of healthy donors) was obtained from Tianjin blood center.

### 3.2. RGD4C-β-Lactamase Conjugate

The RGD4C-β-lactamase conjugate (RGD4CβL, 42 kd) was consist of β-lactamase fused with ACDCRGDCFCG peptide (RGD4C) by recombinant DNA technology. Fusion gene was cloned into *E. coli* BL21 (DE3); the protein was purified with nickel-nitrilotriacetic acid (Ni-NTA) resin, and was further confirmed by western blotting [16,17].

### 3.3. Cell Culture and Animals

C6 cells was cultured in Roswell Park Memorial Institute (RPMI) 1640 medium supplemented with 10% fetal bovine serum (FBS), 100 U/mL penicillin, and 100 μg/mL streptomycin at 37 °C in 5% CO_2_. Wistar rats were purchased from Experimental Animal Center of Academy of Military Medical Sciences (Beijing, China). All animal procedures were approved by the Ethics Committee of Institute of Radiation Medicine of Chinese Academy of Medical Sciences (8 April 2014). The animal studies were conducted in accordance with the regulations of the Ethics Committee of Chinese Academy of Medical Sciences.

### 3.4. Immunofluorescent Staining Assay

The RGD4CβL (0.3 mg) was dialyzed thrice against phosphate buffer solution (PBS), subsequently, 45 μL FITC (1 mg/mL in dimethylsulfoxide) was added, and the mixture was stirred gently for 20 h at 4 °C. The reaction mixture was purified by ultrafiltration, and then washed twice using PBS to remove the unreacted FITC. The target product FITC-RGD4CβL was obtained, and the absorbance at A280 and A495 was measured to determine the concentration of RGD4CβL and the ratio of FITC to the RGD4CβL. The concentration of FITC-RGD4CβL was adjusted and C6 cells were cultured in 24-well plates (4 × 10^4^/well). After washing twice with PBS, the cells were fixed with ethanol at room temperature for 10 min, 0.5 mL serum-free medium and 100 μL of the FITC-RGD4CβL solution were added sequentially. After incubation for 80 min at 4 °C, the fluorescent images were acquired using fluorescent microscopy (DMI 6000B, Leica, Wetzlar, Germany) and the nucleus was stained using DAPI.

### 3.5. Radioactive Technetium Labeling

The RGD4CβL was labeled with ^99m^Tc at its His-tag, as previously described [20,21]. Briefly, [^99m^Tc(H_2_O)_3_(CO)_3_]^+^ was synthesized by adding 1 mL of fresh ^99m^Tc-pertechnetate (10 mCi) from a ^99^Mo–^99m^Tc generator to a mixture of 22 mg sodium borohydride, 4 mg sodium carbonate, and 15 mg sodium potassium tartrate tetrahydrate under atmospheric carbon monoxide, the reaction mixture was maintained in boiling water bath for 20 min. After neutralization using 1 mol/L HCl, [^99m^Tc(H_2_O)_3_(CO)_3_]^+^ was added to a 0.15 mg/mL RGD4CβL solution and incubated for 90 min at 50 °C.

### 3.6. Purification and Radiochemical Purity

The ^99m^Tc-RGD4CβL solution was purified by ultrafiltration using PBS to wash off unbound [^99m^Tc(H_2_O)_3_(CO)_3_]^+^ and passed through a 0.22-μm Millipore filter to eliminate possible aggregates. Thin layer chromatography (detected with an AR-2000 radio-TLC Imaging Scanner, Bioscan, Washington, DC, USA) was then performed to determine the labeling efficiency and radiochemical purity of the ^99m^Tc-RGD4CβL, both directly after labeling and after purification using acetone as mobile phase.

### 3.7. In Vitro Stability

Two portions of 100 μL ^99m^Tc-RGD4CβL were added to 500 μL normal saline at room temperature (25 °C) and 500 μL human serum at 37 °C respectively. Radiochemical purities were assayed using thin layer chromatography at 1, 2, 6, and 24 h.

### 3.8. In Vitro Evaluation of ^99m^Tc-RGD4CβL

C6 cells were implanted in 24-well plates in there logarithmic phase. After overnight incubation, the cells were washed twice using cold PBS, then the ^99m^Tc-RGD4CβL was added at concentrations of 0.02–80 nM. The plates were incubated on ice for 1 h, then washed twice using cold PBS. Portions of 1 mL sodium hydroxide (1 mol/L) were added and the plates were incubated at room temperature for 1 h. The lysates were then collected and radioactivities were measured by a gamma counter (2470 WIZARD2, PerkinElmer, Waltham, MA, USA). Meanwhile, the blocking experiment was performed by adding 50 μg RGD4CβL to the wells and incubating each for 30 min before adding the ^99m^Tc-RGD4CβL.

### 3.9. Blood Clearance of ^99m^Tc-RGD4CβL

Three Wistar rats were intravenously injected with 7 μCi ^99m^Tc-RGD4CβL. Blood samples were collected using a microcapillary at 5, 15, 30, 45, 60, 90, and 180 min after the injection to obtain a radioactivity-time curve. Data were presented as the percentage injected activity per total blood weight (%ID/g). Total blood weight was calculated as 7% of the total body weight.

### 3.10. Biodistribution

The distribution of the ^99m^Tc-RGD4CβL was studied using Wistar rats bearing subcutaneously implanted xenografts of C6 cells. In the present experiment rats bearing C6 xenografts at left armpit were injected with 38 μCi ^99m^Tc-RGD4CβL via the tail vein. At 2, 4, and 8 h post-injection, four rats were anaesthetized, bled, and dissected. Blood, tumor, and normal tissues were weighed, and radioactivities were measured using a gamma counter. Radioactivity uptake was calculated as %ID/g.

### 3.11. Statistical Analysis

Differences in cell binding test were statistically analyzed for each dose point using Student’s *t*-test. Two-sided significance levels were calculated and *p* < 0.05 was considered statistically significant.
